# Microalgae Biomass as a New Potential Source of Sustainable Green Lubricants

**DOI:** 10.3390/molecules27041205

**Published:** 2022-02-11

**Authors:** Leonardo I. Farfan-Cabrera, Mariana Franco-Morgado, Armando González-Sánchez, José Pérez-González, Benjamín M. Marín-Santibáñez

**Affiliations:** 1 Tecnológico de Monterrey, Escuela de Ingeniería y Ciencias, Ave. Eugenio Garza Sada 2501, Monterrey 64849, Mexico; mariana.franco@tec.mx; 2 Instituto de Ingeniería, Universidad Nacional Autónoma de México, Circuito Escolar, Ciudad Universitaria, Mexico City 04510, Mexico; agonzalezs@iingen.unam.mx; 3 Instituto Politécnico Nacional, Escuela Superior de Física y Matemáticas, Laboratorio de Reología y Física de la Materia Blanda, U.P. Adolfo López Mateos Edif. 9, Col. Lindavista, Alc. Gustavo A. Madero, Mexico City 07738, Mexico; jpg@esfm.ipn.mx; 4 Instituto Politécnico Nacional, Escuela Superior de Ingeniería Química e Industrias Extractivas, U.P. Adolfo López Mateos Edif. 7, Col. Lindavista, Alc. Gustavo A. Madero, Mexico City 07738, Mexico; bmarin@ipn.mx

**Keywords:** microalgae biomass, lipids, green lubricants, biolubricants, sustainability, lubrication

## Abstract

Lubricants are materials able to reduce friction and/or wear of any type of moving surfaces facilitating smooth operations, maintaining reliable machine functions, and reducing risks of failures while contributing to energy savings. At present, most worldwide used lubricants are derived from crude oil. However, production, usage and disposal of these lubricants have significant impact on environment and health. Hence, there is a growing pressure to reduce demand of this sort of lubricants, which has fostered development and use of green lubricants, as vegetable oil-based lubricants (biolubricants). Despite the ecological benefits of producing/using biolubricants, availability of the required raw materials and agricultural land to create a reliable chain supply is still far from being established. Recently, biomass from some microalgae species has attracted attention due to their capacity to produce high-value lipids/oils for potential lubricants production. Thus, this multidisciplinary work reviews the main chemical-physical characteristics of lubricants and the main attempts and progress on microalgae biomass production for developing oils with pertinent lubricating properties. In addition, potential microalgae strains and chemical modifications to their oils to produce lubricants for different industrial applications are identified. Finally, a guide for microalgae oil selection based on its chemical composition for specific lubricant applications is provided.

## 1. Introduction

**Figure 1:**
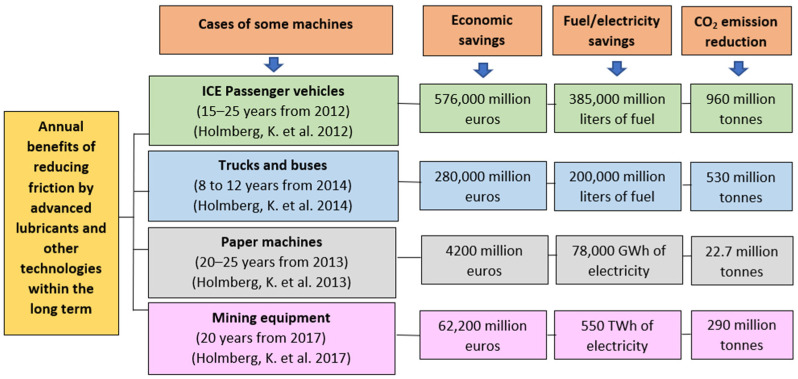
Annual global economic and energy savings, and CO_2_ emissions reduction by reducing friction in different industrial sectors [2,3,4,5].

Lubricants are materials, either in solid, semi-solid, liquid or gaseous state, able to reduce friction and/or wear of any type of moving surfaces. In essence, lubricants are used to form a layer between two rubbing surfaces to separate and protect the surfaces to some extent. Lubricants facilitate smooth operations, maintain reliable machine functions, and reduce the risks of failures while contributing to energy savings. Moreover, the performance and energy consumption of mechanical elements in modern industrial processes, machinery and vehicles depend greatly on the lubricant quality. The service-life of machinery and its components can be increased and energy consumption reduced if the appropriate lubricant is selected for each specific application [1]. According to recent reports [2,3,4,5], significant global economic and energy savings, and CO_2_ reduction can be achieved in different industrial sectors (see Figure 1) by reducing friction losses as well as by using advanced lubricants and tribologically enhanced technology. At present, the increasing prices of crude oil (from around $50 in January 2017 to about $85 in January 2022 for the west Texas intermediate (WTI) crude [6]), the depletion of crude oil reserves and global concern about environmental protection have fostered renewed interest in developing and using lubricants that are renewable, biodegradable, non-toxic, and emit net zero greenhouse gas, i.e., green lubricants.

In general, the requirements of a high-performance lubricant include good tribological properties, high thermal, hydrolytic and oxidation stabilities, high viscosity index and boiling point, low pour point, good corrosion inhibiting properties, etc., which depend on each specific industrial application. Up-to-date, conventional and high-performance lubricants used in industry and vehicles are produced from fossil mineral or synthetic sources blended with additives at different concentrations to attain and modify specific properties. Mineral oils (MOs) are combinations of hydrocarbons in liquid state derived from crude oil through different distillation and refining processes [7], meanwhile synthetic oils (SOs) are obtained by chemical modification of crude oil [8]. Overall, SOs exhibit enhanced thermal and chemical properties as compared to MOs [9]. Then, synthetic lubricants are preferred in contemporary and demanding applications. However, production, usage and disposal of these lubricants have significant impact on environment and health of those who handle them. Hence, there is a growing pressure to reduce demand of non-renewable energy resources and lubricants. In fact, different environmental agencies around the world have introduced restrictions regarding human and environmental toxicity, safety, handling, and disposal of lubricants, which has led scientists to develop upgraded and novel lubricant formulations with higher biodegradation rates than MOs and SOs [10].

According to Willing [11] and Rudnick [12] about 50% of the total amount of lubricants used in the world is incorporated into environment via spillages and evaporation. Besides the difficulties for formal disposal of lubricants, this way of involuntary pollution results in increasing the atmospheric CO_2_ that leads to anthropogenic (i.e., human-induced) global warming. Among the different solutions proposed to overcome this problem, a new generation of biodegradable lubricants produced from renewable vegetable sources is the most promising alternative. Akin to MOs, oleochemicals from vegetable oils (VOs) are disintegrated into CO_2_ and water when disposed into the environment. Nonetheless, the carbon cycle of vegetable oleochemicals is closed, i.e., the amount of CO_2_ released to the environment is equal to the CO_2_ consumed by the crops from the atmosphere, keeping the CO_2_ balance of the atmosphere [13]. Currently, numerous VOs are being explored extensively to produce ecofriendly biolubricants for different industrial applications, since this sort of oils comply with several technical specifications of standard lubricants. In addition, in contrast to petroleum-based lubricants, bio-oils are nontoxic and present various economic benefits such as reduction in energy consumption and labor cost, increased employee safety and improved environmental conditions [14].

Various widespread data compilations about VOs as biolubricants have been recently published [13,15,16,17,18,19,20,21,22,23,24,25], which cover a significant amount of research work addressing this initiative in the last decades. The great acceptability of VOs for producing alternative biolubricants is reflected on the fact that global companies such as BP PLC, Chevron Corp, ExxonMobil, Repsol, Royal Dutch Shell PLC, Total and FUCHS, among others, have recently produced and marketed some of these lubricants. Moreover, it is expected that the market for biolubricants increases at an annual growth rate (CAGR) of 4.25% during the period between 2019 and 2024 [13,14]. Thus, many more entities are investing in the development of new and more advanced biolubricants for different sectors in industry.

The most promising and explored VOs for biolubricants are obtained from edible crops as sunflower, rapeseed, soybean, palm, palm kernel, coconut, canola, olive, castor and mustard, as well as from some inedible crops as Jatropha, Callophyllum inophyllum and neem [13]; being these last preferred because they do not compete with production of food crops [26]. However, even using inedible bio-oils for lubricants production is auspicious only if there is enough land area for cultivating edible and inedible crops to satisfy the food market demand. Furthermore, considering that also biofuels are being produced using VOs [27], conflict of arable lands will still persist [28]. To tackle this problem, researchers have proposed the use of neat VOs not as full biolubricants, but in blends at low proportions (≤20%) with MOs or SOs [29,30,31,32,33,34]. This strategy has been partly fruitful and has resulted in improved lubricating properties of MOs while keeping biodegradable a significant proportion of the lubricant; but poor cold flow properties and oxidation stability of VOs are still limiting factors to achieve high-performance [35,36,37,38,39,40]. Despite these limitations, the use of neat VOs to produce biolubricants is acceptable for applications where high-performance is not demanded or when the lubricant is used only for short periods and/or at room temperature (20–35 °C). Chainsaw oils, drilling muds, bicycle chain mechanism, etc., are some examples of this kind of short-term applications.

An emerging alternative to limited arable land and to achieve a sustainable production of biofuels and biolubricants is based on microalgae biomass production and exploitation. Microalgae are microscopic unicellular microorganisms that grow in marine and freshwater environments. They can convert nutrients, either in an artificial medium or wastewater, into biomass with a wide range of possible high-value cellular constituents [41]. Among the most potential uses and products of microalgae biomass are CO_2_ mitigation [42,43], agro-industrial and wastewater treatment [44,45], biogas upgrading [46,47,48,49], animal and human food supplements [50,51,52], pharmaceutical products [53], cosmetics [54], pigments and carbohydrates [55,56], proteins [57], vitamins [52], fertilizers [58], and biofuels [59,60,61,62]. The last, which are the most related product to biolubricants, have received a great attention and have reached significant progress in the last decades, demonstrating its reliability and acceptability in the industrial sector. A similar future trend is expected for microalgae-based biolubricants.

Biofuels and biolubricans share a common origin, that is, they are both derived from fatty acid esters found in vegetable oils or animal fats. So, existing studies on microalgae-based biofuel production may shed some light into the less explored field of biolubricants. However, considering that biofuels are substances expected to release energy in a controlled manner to generate mechanical work in engines and biolubricants are intended to reduce friction at sliding interfaces, different properties must be procured for each product by chemical treatments. In the case of biofuels, certain cetane number (typically higher than No. 2 in diesel) and low viscosity (similar to conventional diesel at most) are some of the most important properties, which are basically induced by a single transesterification reaction [63]. On the other hand, different properties such as lubricity, low pour point, high thermal stability, particular viscosities, etc., are expected for biolubricants. Hence, a variety of chemical treatments of the fatty acid esters are needed to obtain good performance biolubricants. The main biolubricant properties can be attained by conducting a second transesterification, or by other conversion techniques, namely, epoxidation, hydrogenation, etc., including a further formulation with additives [13]. These techniques are discussed in Section 4.2.

The advantages for production of some species of microalgae over land crops are their easy growth in a variety of environments with different photosynthetic efficiency, the fact that they require smaller lands and lower water consumption for cultivation, as well as their ability to produce substantial amounts of lipids [59,60,61]. These advantages can be exploited in lubricants production if microalgae oils with appropriate tribological and physicochemical properties are identified. It is noteworthy, however, that the use of microalgae to produce oils with high-valuable fatty acids for lubricants is inevitably linked to the concept of algal biorefinery as a sustainable approach to valorize algal-based biomass towards multiple product recovery [64,65]. Therefore, this review paper has four main purposes, the first is to outline and discuss the attempts and progress on microalgae bioproduction technologies and methods for developing potential bio-oils as lubricants for different applications. The second is to identify the most suitable prospective microalgae strains for production of biolubricants according to their lipids accumulation ability, fatty acids profiles and large-scale production feasibility. The third is to review the main chemical modifications required to enhance lubricant properties. The fourth and final is to provide the reader with a guide to select microalgae oils and their chemical modifications for producing biolubricants with specific industrial purposes.

## 2. State-of-the-Art of Microalgae-Based Biolubricants

In contrast to the significant development and research on microalgae biomass for biofuels production [59,60,61,62], its application in biolubricants have received limited attention. To our knowledge, few works have been published about the production, exploration and enhancement of the performance of some microalgae oils as biolubricants for industrial applications, but its number is expected to grow in the near future due to the green advantages of microalgae over other sources. Xu et al., [66] and Xu et al., [67] evaluated and improved the tribological performance of a bio-oil from Spirulina microalgae. These authors prepared the bio-oils by hydrothermal liquefaction followed by an esterification process to decrease corrosiveness of the sliding metals and improve lubricity. The authors reported acceptable tribological behavior for the crude bio-oil, which was enhanced in the esterified microalgae oils. Dziosa and Makawska [68] proposed a method for preparing a lubricant from the biomass of single-cell green algae Chlorella sp. grown in laboratory. The method was based on dehydration of the biomass obtained from the culture, followed by freeze-drying and solvent extraction to obtain the lipids. The resulting lubricant exhibited similar chemical structure and viscosity-temperature properties to rapeseed oil. So, the authors proposed this microalgae oil as a suitable replacement for rapeseed oil biolubricants. Xu et la., [69] evaluated the tribological behavior of steel/steel pairs under lubrication with suspensions of graphene/MoS_2_ in esterified bio-oil with different mass ratios, loads and rotating speeds. The bio-oil was made from crude Spirulina microalgae via catalytic esterification. The authors reported a synergistic lubricating effect of graphene and MoS_2_ at contents of 0.5 wt%, which resulted in a reduction of CoF and wear of the steel specimens under boundary lubrication conditions. Later, Xu et al., [70] evaluated the lubrication effect of an esterified Spirulina microalgae bio-oil with dispersed graphite on steel/gray cast iron friction pairs. They found that high loads and low sliding velocities contributed to good tribological performance of the friction pairs. On the other hand, Xu et al., [71] prepared Chlorella and Spirulina microalgae oils via co-liquefaction under sub- and supercritical ethanol conditions, which were used as partial substitutes for a CD SAE 15W-40 engine oil. The friction and wear behavior of the oils were tested in a four-ball tribometer and the best lubricating behavior was observed when the weight contents of the bio-oils in the engine oil was 10%. Recently, Cheah et al., [72] produced an oil derived from dried Chlorella biomass and modified it chemically as biolubricant via catalytic esterification. They evaluated the tribological behavior of the modified microalgae-based bio-lubricant in hydrogen-powered engine applications and compared it with the performance of a SO (poly-alpha-olefin oil (PAO)) and different blends of PAO with the biolubricant (1, 2, 5, 10, and 20 (*v*/*v*%)). All the blends generated better lubricity than neat PAO, which proved these modified microalgae oils as potential biolubricants for this application. Finally, the growing interest on developing microalgae-based lubricants at a commercial scale is reflected on a recently patented method for manufacturing algal oils [73].

The aforementioned research reports demonstrate that microalgae oils obtained from different strains and with appropriate chemical modifications can exhibit suitable properties as lubricants for some industrial applications. However, considering the numerous available microalgae strains and their variations in terms of cellular constituents, extensive research is required to identify the more promising microalgae strains and cultivation conditions, their harvesting and oil extraction conditions and further chemical modifications leading to the production of oils with suitable chemical composition and physicochemical properties as lubricants. In the following, the main steps in microalgae oil production are described.

## 3. Microalgae Oil Production

Although microalgae lubricants are potential green substitutes for MOs, SOs, and other VOs, microalgae oil production is still not feasible at a commercial level due to the low biomass concentration achieved by most production methods. Nevertheless, these can become viable by designing efficient photobioreactors, low-cost and effective techniques and technologies for biomass cultivation, harvesting and oil extraction/purification. Furthermore, the enhancement in obtaining precursors of biolubricants can be also realized by genetic engineering tools to direct the metabolic pathways of microalgae to the desired high lipid production [74]. In general, the microalgae oil production process involves four steps: cultivation, biomass growth/lipids accumulation, biomass harvesting and oil extraction/purification, which are discussed next.

### 3.1. Cultivation (Photobioreactors and Growth Conditions)

**Figure 2:**
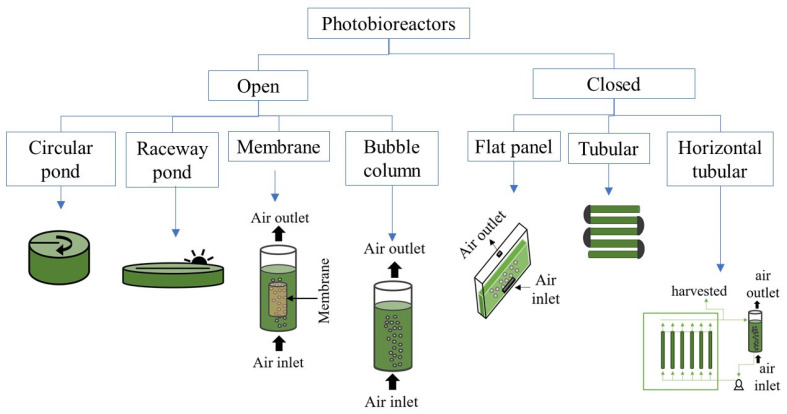
Classification of most common photobioreactors according to their configuration.

In microalgae cultivation is important to consider and control different variables, namely, light supply, photobioreactor type, dissolved oxygen concentration, culture media, etc., to optimize their growth and byproducts accumulation. Microalgae metabolism is versatile since they are able to grow under heterotrophic, autotrophic and mixotrophic conditions, as well as to tolerate a broad range of pH, salinity and temperatures [75,76,77,78], among other conditions. Large amounts of microalgae can be produced through various configurations of photobioreactors (either close or open), under different environmental conditions (outdoors or indoors) and light regimes (light/dark cycles). Figure 2 shows a classification of the most common photobioreactors according to their configuration. In close photobioreactors are usually made up of glass or plastic tubes which allow the control of water evaporation and minimize contamination risks. These photobioreactors can be placed under outdoors or indoors conditions depending on the product of interest. The surface/volume ratio of a closed photobioreactor is up to 80 m^−1^, which promote significant biomass concentrations [79]. The microalgae growth in closed photobioreactors is limited by accumulation of O_2_ and the proper supply of CO_2_. Therefore, it is necessary to determine the appropriate length of the tubes since it determines the residence and mixing times of the culture broth inside of the photobioreactor [80,81]. Mixing is necessary to homogenize the culture and distribute all the nutrients, as well as to avoid light limitation. In close systems, mixing is attained by air/CO_2_ supplying [82]. On the other hand, open systems can be natural lakes and ponds, artificial circular and raceway ponds, etc. [83]. Open ponds or high-rate algal ponds (HRAP) have been widely used for growing microalgae at full-scale due to higher technical and economic feasibility as compared to closed photobioreactor configurations [84]. The main variable in HRAP systems is the total area employed for their installation. They consist of a pond divided into two or four channels with depth in the range from 0.2 to 0.4 m, which promotes penetration of light inside the photobioreactor [85]. In contrast to closed systems, mixing in open systems is achieved by mechanical arms or paddle wheels. The CO_2_ supply is vital to promote growth of biomass and to foster O_2_ desorption, which may affect the productivity of the biomass and the product of interest [86,87]. Unfortunately, the risk of contamination by other microorganisms increases in open ponds due to exposure to environmental conditions, meanwhile significant water losses due to evaporation are generated.

**Figure 3:**
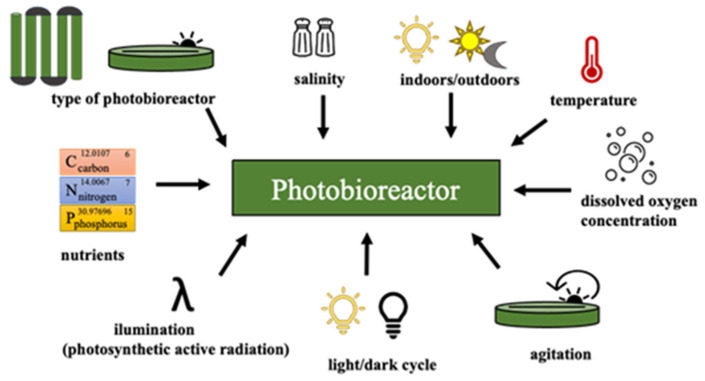
Schematic representation of the main variables in cultivation of microalgae biomass.

To enhance the growth of microalgae biomass is necessary to supply all the macro-nutrients (C, N, P, K, etc.) and light together in an appropriate mixing to the culture broth [88,89,90,91]. Biomass productivity is also function of light intensity and its wavelength. The increase in light intensity helps to improve biomass growth until light saturation is reached, which afterwards, causes negative effects on growth rates (photoinhibition) [89,90]. Overall, the main variables to consider for cultivation of microalgae biomass are represented in Figure 3. Changes in some of them induce different conditions or stress factors that determinate the rate of growth, biochemical reactions, and hence, different byproducts accumulation, such as proteins, carbohydrates, pigments and lipids [91]. Thus, changes in culture conditions, nutrients deficiency and physical conditions are the most important strategies deployed in microalgae cultures to promote the production of certain valuable compounds, as high-value lipids/oils for biolubricants.

A outstanding characteristic of microalgae biomass is that they can grow in undefined and atypical culture media, namely, freshwater, brackish water, seawater, and wastewater due to their ability to metabolize both organic and inorganic nutrients. The use of these kind of culture media, wastewater in particular, has been successfully applied to meet a circular economy and reduce cost of microalgae cultivation [92]. The use of digestate from anaerobic digestion process [47,93], brewery wastewater [94], agroindustrial wastewater [95], etc., are examples of sources of culture media alternatives for microalgae production under circular economy schemes.

In addition, microalgae have the ability to grow under autotrophic, heterotrophic and mixotrophic conditions. Heterotrophic and mixotrophic conditions exhibit higher biomass yield, extended exponential phase of growth, and less biomass loss [96]. Under mixotrophic conditions, both inorganic and organic carbon can be assimilated under certain operational conditions to increase biomass productivity and other high-value byproducts [97,98]. For example, Kujawska et al., 2021 [99], demonstrated waste glycerol as a potential organic carbon source to reach a *Schizochytrium* sp. biomass productivity of 48.85 ± 0.81 g/dm^3^, producing docosahexaenoic acid at a concentration of 21.98 ± 0.36 g/dm^3^.

Another strategy to improve biomass productivity and obtain higher concentrations of byproducts is the immobilization of microalgae biomass. Benasla and Hausler 2021 [100] demonstrated the effect of immobilized green *Raphidocelis subcapitata* microalgae in alginate gel to obtain a high lipid productivity for biodiesel production. In addition, Savvidou et al., 2021 [101] immobilized *Nannochloropsis oceanica* and *Scenedasmus almeriensis* cells by enzymatic (cellulase) and mechanical (glass beads) treatments, generating protoplasts as a means of incorporation of magnetic nanoparticles. The magnetic properties supported the successful immobilization and growth of microalgae cells on a vertical magnetic surface exposed to light and without any supplement. It allowed a considerable increase of biomass productivity.

### 3.2. Lipids (Oil) Accumulation Operational Strategies

The main classes of lipids found in microalgae are membrane lipids (glycosylglycerides, phosphoglycerides and betaine ether lipids) and storage lipids as triacylglycerol [102]. The amount of lipids in a microalgae strain depends on operational conditions of the culture broth. Triacylglycerids (TAG) are the main lipids in microalgae strains, they are used for storing energy and are the product of the primary metabolism [103]. Under light conditions, microalgae fix carbon via Calvin cycle and produce different molecules, including TAG, and depending on the specie, photobioreactor configuration and stress operational conditions, TAG contents can be increased in microalgae cells [104]. TAG are the most common neutral lipids and can be found in cell cytoplasm. They are formed when three similar fatty acid (FA) molecules are attached to glycerol, which serves as backbone for the molecule [26]. TAG is the base composition (92–98%) of biolubricants and their structure characteristics and concentration are known to determine the main lubricant properties. Therefore, the identification of a strain and growth conditions for accumulating the largest amount of lipids with triglycerides having certain FAs is the goal.

The concentration of TAG during “normal” microalgae growth (no stress) is usually small, but this concentration increases remarkably under stress conditions by various strategies as [105,106]: (1) deprivation of nutrients such as nitrogen and phosphorous; (2) oxygen saturation; (3) light intensity and illumination/dark cycles; (4) salinity; (5) temperature; and (6) mutant genes. Table 1 describes the main stress conditions to promote TAG accumulation in microalgae cells.

### 3.3. Microalgae Biomass Harvesting

Harvesting consists of separating microalgae from its growing medium. Large-scale production of microalgae biomass and its ulterior extraction is the main challenge to develop cost-effective technologies for efficient harvesting and byproduct extraction [121]. In this sense, different harvesting technologies have been assessed to enhance the productivity of byproducts and, especially in this case, lipids content.

**Figure 4:**
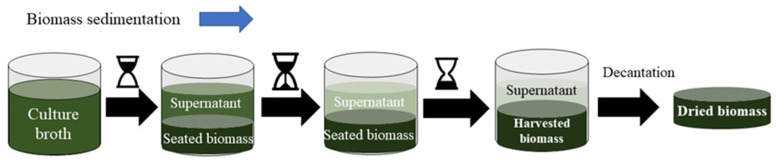
Schematic representation of the sedimentation technique to harvest microalgae biomass.

Before applying a harvesting method, it is necessary to consider the type of microalgae strain and the operational conditions of their growth, as well as the density, size and the physicochemical characteristics of the byproducts [122]. Harvesting of microalgae can be divided into two step processes. The first consists in separating microalgae biomass from the culture broth (2–7% dw), the second step is the thickening to promote more concentrated biomass than in the first step [121]. All harvesting processes may include thickening, dewatering and drying. Figure 4 shows a representation of a green harvesting method based on sedimentation. It is one of the simplest and cheapest green techniques to separate the microalgae from culture media. However, harvesting can be also based on chemical, physical, biological and magnetic methods. Table 2 summarizes the characteristics of the different methods used to harvest microalgae biomass.

### 3.4. Microalgae Oil Extraction

**Figure 5:**
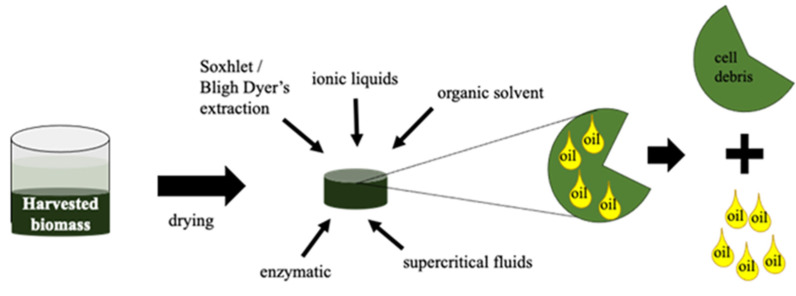
Schematic representation of oil extraction from microalgae biomass.

The next step after biomass harvesting is the application of an efficient lipids/oil extraction technique. This may be carried out by different physical and chemical methods, as represented schematically in Figure 5. The selected method should be fast, easily scalable and effective without causing chemical changes to the extracted lipids [132]. Prior to oil extraction is necessary to apply a pre-treatment method in the cell biomass. Cell disruption is the most common pre-treatment method and depends on the characteristics of the biomass [132]. Bead beating, autoclaving, grinding, osmotic shock, homogenization, freeze drying and the addition of ≥10% (*w*/*v*) NaCl are other methodologies for cell disruption [121,122,132]. Table 3 summarizes the characteristics of the most common oil extraction methods from microalgae biomass. Residues generated after oil extraction are rich in different byproducts, such as carbohydrates and proteins, that may be used to make other different products as biogas or biohydrogen, food additives, pigments, fertilizers, etc. [121,133]. Considerations of microalgae oils as lubricants are provided in the next section.

## 4. Considerations of Microalgae Oils as Lubricants

To identify a microalgae oil as a potential lubricant, it is important to know its chemical characteristics, the lubricating properties required for the specific applications and strategies to enhance those properties. Most of studies regarding the relationship between chemical structure of oil and lubricating properties, and the strategies for lubricant properties enhancement have been focused on edible and non-edible VOs. However, considering the similarities in chemical composition between microalgae oils and vegetable oils, the knowledge previously gained in the study of VOs can be also applied to microalgae ones.

### 4.1. Relation of Oil Chemical Structure with Its Lubricating Properties

In general, any neat bio-oil, including microalgae oils, is composed by 92–98% of TAG in combination with a variety of FA molecules attached to a single glycerol structure [12]. Depending on the triglyceride structure and FAs contained, different lubricating properties, namely, lubricity, viscosity, pour point and oxidation stability can be expected. The key chemical factors influencing the aforementioned lubricant properties of VOs are: (1) the carbon chain length; (2) the type of FAs (saturated fatty acids (SFA), monounsaturated fatty acids (MUFAs) and polyunsaturated fatty acids (PUFAs)); and (3) polarity [13,137]. The corresponding characteristics of the chemical factors influencing the main lubricating properties of bio-oils are compiled in Table 4.

#### 4.1.1. Lubricity

Lubricity of bio-oils is principally influenced by FAs unsaturation, chain length, branching, and polarity. The CoF is expected to decrease with increasing the carbon chain length since longer hydrocarbon chains of FAs produce stronger molecular linkages. In addition, longer FA chains form thicker adsorbed films on surfaces. On the other hand, decreasing the degree of branching in the base oil enhances lubricity. So, branched-chain acids are more prone to produce wear than linear-chain acids with the same carbon number [139]. Different FAs in bio-oils have significant impact on the lubrication efficiency. It has been demonstrated that increasing the degree of unsaturation of FAs diminishes wear performance [140,141]. Thus, PUFAs promote weak protective tribo-films. Polar structures are also believed to provide good boundary tribo-films. The straight physical contact between oil molecules and metal surfaces is created by the chemical interaction between the non-polar end of metals with the polar functional groups from the oil producing a layer that separates the rubbing surfaces [143]. So, microalgae oils with increased polar functionality could exhibit superior boundary lubrication (BL) properties than MOs due to stronger adsorption on the metal surfaces. Then, microalgae oils may be useful in applications involving BL such as in engines, power transmissions, metal working/machining, gas/petroleum drilling, compressors, turbines, etc.

#### 4.1.2. Viscosity

Viscosity is a measure of the resistance to flow or internal friction when a fluid is subjected to shear stresses. The fluid viscosity depends on its composition, shear deformation, temperature and pressure. Based on the flow and viscosity curves (rheograms) reported in the literature, bio-oils behave mainly as Newtonian fluids [149,150,151]. Viscosity of bio-oils is also influenced by carbon chain length and FAs type. High viscosity promotes high resistance to flow, thicker lubricant films and increased power consumption, while low viscosity means low flow resistance, thinner lubricant films and reduced power consumption. Depending upon each specific application, a certain lubricant viscosity grade is required. For example, high-viscosity oils are demanded for gears operating under high loads for achieving stronger and larger lubricant films, meanwhile low-viscosity oils are needed for modern engines to reduce fuel consumption [152,153]. In addition, high viscosity is appropriate to reduce wear in machinery, but it is negative for friction and power saving. Thus, both low and high-viscosity bio-oils can be useful. Viscosity of bio-oils increases with the length of the FA chain, which is due to the growth of the degree of random intermolecular interactions [145]. Hence, oils with long chain FAs usually have high viscosity while oils with short chain FAs poses low viscosity. Furthermore, the degree of unsaturation is also an important factor contributing to oil viscosity; a single double bond increases viscosity, whereas two or three double bonds decrease viscosity [146]. On the other hand, it is well known that viscosity decreases with temperature (at a constant pressure) and increases as a function of pressure for a given temperature. This dependence on pressure is a necessary characteristic of lubricants to achieve effective elastohydrodynamic lubrication, but its effect in sliding frictional zones and hydraulic systems is to increase energy consumption due to high viscous dissipation. If the temperature dependence of viscosity is investigated, keeping shear deformation and pressure constant, the temperature-viscosity coefficient may be obtained and related with thermal stability. If bio-oils are exposed to heat or high temperatures (thermal oxidation process), their physicochemical properties are modified by alteration of FA chains, which may produce deposits in the form of varnish, carbons, or particulates [150,151,154,155]. In addition, the viscosity-pressure relationship behavior must be considered if the oil will be used for low or high load applications. This relationship can be fitted to some empirical models, from which the pressure-viscosity dependence coefficient is obtained, this parameter usually decreases with increasing pressure but increases with increasing pressure above an inflection value [149,150,151,155]. Finally, when bio-oils are used as matrixes for dispersed micro or nanoparticles, the viscosity of the lubricant may become shear dependent (non-Newtonian behavior) with increasing particle concentration, then shear stability, namely, viscosity reduction as a function of shear or shear-thinning, should be investigated [152,154,156]. When flowing, non-Newtonian shear-thinning lubricants can decrease power consumption.

#### 4.1.3. Pour Point

Pour point refers to the lowest temperature at which the lubricant loses flow capability and becomes semi-solid matter. Pour point (to be low) is the most important low-temperature property wanted in lubricants employed at extremely low temperatures. It is known that the pour point of bio-oils decreases as the number of double bonds in the molecules increases. Oils with high content of unsaturated FAs chains will exhibit lower pour points because the FAs chains bent in a molecular arrangement that prevents their close packing when cooling [147]. Then, a higher degree of unsaturation is positive for low-temperature properties.

#### 4.1.4. Oxidation Stability

Oxidation of lubricants occurs by a chemical reaction with oxygen. It is facilitated by high temperatures, high pressures and exposure to water and other contaminants (i.e., debris and soot). Thus, the oxidation stability of a lubricant refers to its capacity to withstand oxidation. High oxidation stability is demanded for lubricants, mainly for those exposed to harsh operating environments at high temperatures for prolonged periods (i.e., engines, turbines, compressors, etc.). The principal consequences of thermo-oxidation in bio-oils are polymerization with further increase in viscosity, reduction of viscosity index and alteration of lubricity [29,150,151,154,155]. Oxidation stability is determined by the dominant FAs contained in the oils. The presence of PUFAs, namely, linoleic and linolenic acids, in large proportions leads to high rates of oxidation because double bonds in the alkenyl chains react easily with oxygen to form free radicals that lead to polymerization of oil and further disintegration [148]. Hence, a lower unsaturation degree is good for achieving higher oxidation stability, but it is negative for low-temperature properties, in particular, for the pour point.

### 4.2. Chemical Processes for Improving Lubricant Properties of Microalgae Oil

The production of fatty esters [157,158] and estolides [23,157,159] from neat VOs by chemical conversion processes has gained great attention because these enhance lubricity and thermal stability properties significantly. Basically, fatty esters are produced from the combination of a FA with an alcohol-like by transesterification processes. Mostly, they have a concentration of 98% of methyl esters of long chain FAs and the rest comprising free glycerin; mono-, di-, and triglycerides; antioxidants; sterols; phospholipids; and water [160]. Methyl and ethyl esters of FAs are the most popular biolubricants (oils and greases) for automotive applications, marine engines, compressors, hydraulic systems and gears [158].

Estolides are a developing class of natural and synthetic compounds synthesized from hydroxy oils or by the condensation of FAs through the olefin of a second FA. Estolides exhibit good oxidation resistance, which is a common drawback of VOs [23,161,162]. In addition, oleic acid-based backbone estolides present the best-performing properties for lubricant applications [23,157]. In addition, other chemical conversion processes for achieving these improved VO derivatives are transesterification, partial hydrogenation and epoxidation [13,163].

Transesterification is a reaction catalyzed generally by acids or bases. It is produced between an ester and an alcohol where the alkoxy group of the ester is replaced by alcohol and vice versa [27]. It can be classified into acid-catalyzed or base-catalyzed transesterification according to the type of catalyst employed. In addition, the complexity of the final ester contributing to some properties of the biolubricant is dependent on the alcohol used for the alkyl interchange. The catalyst removal from the final product is easier if alternative heterogeneous catalysts are used [63].

Hydrogenation is a chemical process involving the addition of hydrogen to the C=C bonds in the triglycerides of an oil molecule [164]. The process generates the simultaneous saturation of double bonds, positional isomerization and geometric (cis-trans) isomerization. It is commonly carried out in the presence of a support or Raney Ni catalyst at temperatures from 150 to 225 °C and pressure from 69 to 413 kPa, which generates undesirable toxic Ni traces in the oil [165,166]. So, partial hydrogenation can be a more suitable alternative. It is attained by selecting an appropriate catalyst to reduce linolenic acid before linoleic and oleic acids. This process has been demonstrated to prevent deterioration of the pour point of biolubricants [164,167].

Epoxidation occurs by the reaction of double bonds by peroxy acids and removal of the C=C bonds using conventionally acid ion exchange resins, enzymes and metal catalysts [168]. In industry, the most common epoxidation technique for the production of improved VOs is the Prilezhaev process, in which double bonds are modified to obtain new value-added chemicals or monomers for polymers [169]. Considering the inherent drawbacks of this process, namely, low epoxide selectivity due to oxirane ring opening and corrosion issues, many efforts have accordingly been made for developing new catalytic systems to produce epoxidized VOs in a more selective and efficient manner [170].

Apart from chemical conversions, microalgae oils may be improved for lubrication purposes by incorporating chemical or physical additives. Additive technology has been extensively studied for conditioning different lubricant properties of VOs and their derivatives (esters and estolides) for meeting specific demands. Additives are classified according to the property to be improved in the oil. Commonly, a formulated oil consists of an oil base stock blended with different additives at a concentration around 5%wt. Different antioxidants, detergents and dispersants, viscosity modifiers, corrosion inhibitors, pour point depressants, extreme pressure and anti-wear additives, including nanoparticles, are the most used to condition diverse VOs [13]. The main effective additives used and reported for producing biolubricants are shown in Table 5. It is important to highlight that biolubricants based on esters or estolides formulated with additives have demonstrated superior lubricity as compared to neat VOs or blends with MOs or SOs [157,171]. In addition, since VOs have high solubilizing power for polar contaminants and additive molecules, the formulation can be relatively easy. However, it should be noted that inclusion of additives that contain oxygen and nitrogen, which are the majority, in VOs could interfere with the capability of the esters to adhere to the metallic surfaces promoting negative influence on lubricity. It is because the additives having its own polarity will contend with the surface-active compounds [172]. Thus, additives should be selected and incorporated carefully based on chemical compatibility and synergistic effects.

Biodegradability is the most important characteristic regarding the environmental fate of any substance and product. In general, bio-oils are more readily biodegradable than MOs and SOs, as reported by various research groups that have investigated the biodegradability of sunflower oil, colza oil, castor oil, and oleic esters either in water or soil [185,186,187,188,189,190]. Nonetheless, it is important to note that any chemical conversion and additives inclusion to oils may modify its biodegradability as compared to neat oils. Although these chemical modifications help to improve lubricant properties, they may reduce biodegradability to some extent. For example, Luna et al., 2015 [188], performed a comparative study of biodegradability of neat castor oil, esterified castor oil and MO in an aqueous environment by aerobic microorganisms. They found that esterification caused an alteration of the half-life time of castor oil. Neat castor oil had a half-life time of 12 days meanwhile esterified castor oil was increased to 20–30 days. Despite the increased half-life time of castor oil due to esterification, biodegradability remained significantly lower than that of MO (200 days half-life time). As for the effect of additives in bio-oils on their biodegradability, there is a lack of research studies. Eisentraeger et al., [185] and Hahn et al., [186] compared the biodegradability of different neat ester-based lubricants with and without additives (unknown concentrations and compositions). Both groups found that biodegradability was similar with and without additives. Considering the very particular conditions and formulations evaluated in these reports, an overview about the effects of additives on biodegradability of bio-oils is still not available. So, upgraded and certified modern biolubricants based on microalgae oil should comply with biodegradability studies regarding the specific formulation (neat or modified base oil + additives).

## 5. A Guide for Selection of Microalgae Oils for Producing Biolubricants for Different Applications

**Figure 6:**
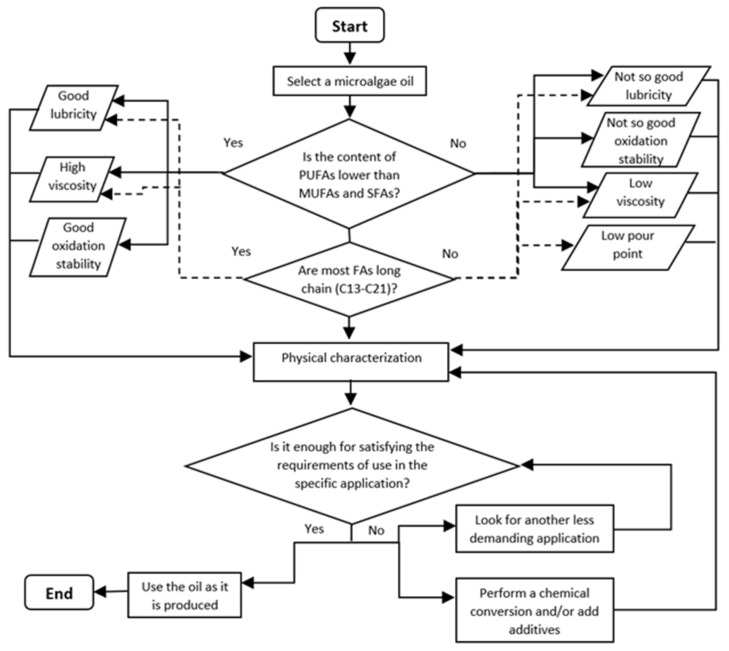
Flow chart for selecting microalgae oils for bio-lubricants production.

Overall, considering the above compilated information about the characteristics and production of microalgae oils and their possible optimization through chemical treatments, a general procedure is suggested to select suitable microalgae oils to produce biolubricants for different applications. The procedure is illustrated in the flow chart of Figure 6. The first step is to select a microalgae oil from a strain able to produce a significant amount of lipids containing a high concentration of FAs under feasible conditions. In addition, it must be considered that the selected microalgae strain has the required biomass productivity and growth conditions for full-scale production. Otherwise, the microalgae biomass production process must be conditioned, or another strain selected. Depending on the FAs type and concentration documented in the microalgae oil, the most important lubricant properties (lubricity, viscosity, pour point and oxidation stability) can be qualitatively predicted. These properties should be promising to meet the requirements of the desired specific application and then the oil must be fully characterized to determine the lubricant properties precisely. If the oil properties comply with the requirements for the target application, the oil can be used as biolubricant as it is produced. Should the application requirements be more demanding, the microalgae oil must be improved by chemical conversion and/or aggregating additives. In case modifications are not effective, other application should be sought for the biolubricant.

There are numerous reports in literature about different microalgae strains producing lipids/oils containing significant amounts of FAs, which can be suitable for biolubricants. A compendium of the most significant and well-documented information of microalgae strains with potential to produce biolubricants is summarized and provided in Table 6; it includes biomass productivity, lipids, FAs concentrations, and production parameters.

According to the data reported in Table 6, the oils from microalgae were classified in terms of their FAs composition to suggest potential biolubricant applications, as given in Table 7. The qualitative relationship between saturation or unsaturation level and chain length given in Table 4 was used for making the classification and ranking. The ranking of the properties was established only according to the comparison between the reported microalgae oils presented in Table 6. In addition, the possible applications suggested were considered from available commercial biolubricants. The selection of the possible applications for microalgae oils was based on the main qualitative requirements of lubricity (low/regular performance or high-performance), viscosity (low or high), pour point (low temperature operation) and oxidation stability (high temperature operation) of each specific application.

## 6. Challenges in the Microalgae-to-Biolubricant Production and Use

In general, the main strengths of the lubricants production from microalgae biomass are the sustainability and eco-friendly characteristics of microalgae production and the feasibility to control the amount and type of favorable fatty acids in the oil through controlling microalgae growth operational parameters. Meanwhile, its main weakness so far are production scalability issues. Once promising microalgae oils have been selected for a specific application, the next step is the full-scale production of the strains (preferably using waste sources). Here, it should be considered that the efficiency of microalgae and biolubricant production comprises various challenges. In general, lipids (7–23%), proteins (6–71%), and carbohydrates (5–64%) compose the microalgae biomass with proportions that depend on the specific algal species and growth conditions. Some microalgae strains can accumulate more than 50% lipids by dry weight, but special stress conditions must be applied for effectively producing TAG with certain FAs at high concentrations. This requires extensive research in laboratory and even more in field. In the case of microalgae biomass production, some requirements should be faced, as the use of sanitary raw materials for cultivation and growth, and assurance of unialgal culture (no-contaminants/other cultures) in photobioreactors. To produce biolubricants, clean lipids/oils extracted from the microalgae biomass are needed. Then, efficient harvesting and oil extraction techniques must be applied according to the species, growth stage and lipid content in the biomass. These requirements make production scalability problematic, since harvesting and oil extraction methods are still expensive and not applicable for large-scale production. Up to date, the large-scale commercialization of microalgae biolubricants is limited by the lack of research on production of specific microalgae biomass with high-value lipids for biolubricants, which opens a research gap for the coming years. According to da Silva and Reis [200], other open problems for the commercial large-scale microalgae production are:

- Design and construction of photobioreactors (open and close). Deviations from design specifications of photobioreactors due to little experience of construction companies and biologists on these new facilities cause substantial delays to the inception of microalgae biomass production projects.
- Harvesting, seed culture preparation and inoculation for scales up to 300,000 L of microalgae. Lack of skills in handling microalgal cultivation, harvesting and oil extraction operations in large scales is a considerable obstacle for efficient operations.
- Climate changes and environmental and land use regulations. Site specific problems such as unexpected power and water outages, seepages, contamination, water evaporation, drastic weather variations and requirement of special land use permissions can cause production stops, or even, death of the culture broth.
- Assuring product quality and consistency by using green processes. Daily culture sampling and analyses is required to assure consistency and quality of the product. Reaching an acceptable quality and consistency of the product by incorporating green processes for harvesting and oil extraction is currently a research challenge.
- On the other hand, akin to other VOs considered as potential candidates for biolubricants production [24], some challenges must be overcome to use confidently and extensively microalgae biolubricants for industry and vehicle applications. The main challenges are:
- Guaranty homogeneity and continuous availability of the product. It depends on the supplier, feed stocks, and production methods, which is line with the microalgae-based oil production challenges.
- Guaranty of proper compatibility with machine materials [201,202,203], acceptable thermal oxidation stability and cold weather operation. These properties must be further evaluated and improved for each oil/lubricant meeting with stringent specifications before marketing.
- Guaranty of acceptable biodegradability and low toxicity of microalgae biolubricants in case of chemical modification of the base stock oils. Biodegradability of the biolubricants can be worsened by chemical modification and additivation. It must be further evaluated and treated to guaranty the eco-friendly characteristics.
- Acceptance by machine manufacturers. Machine performance, emissions, durability, and biolubricant oxidation in a wide range of machines and sizes need to be demonstrated feasibly and widespread to increase consumer and manufacturer confidence. In addition, the environmental benefits offered by microalgae oil over petroleum lubricating oil, or even VOs, need to be popularized.

Finally, should the above challenges be overcome, microalgae oils can emerge as the most sustainable and eco-friendly sources to produce advanced biolubricants for different industrial applications.

## 7. Conclusions

Microalgae oils are more sustainable candidates for production of biolubricants than other already used edible or non-edible VOs. In comparison to conventional petroleum-derived lubricants, VOs and microalgae oils lubricants are cleaner, renewable and non-toxic, which make them ecofriendly. Nonetheless, VOs production competes for the utilization of arable land used for cultivation of edible feedstocks, which can affect the food supply chain when full-scale production of biolubricants is required. In contrast, microalgae can be growth in different environments with different photosynthetic efficiency and require smaller lands and lower water consumption for cultivation and production of considerable oil contents while addressing other environmental benefits. Various microalgae oils have similar chemical composition to VOs in terms of different fatty acids, which evidences their potential as biolubricants. In contrast to VOs, FAs and oil accumulation in microalgae can be readily conditioned through the variation of cultivation and growing parameters such as photobioreactor type (close or open), salinity, temperature, nutrients, dissolved oxygen, illumination, light/dark cycles and agitation.

The main strengths of the lubricants production from microalgae biomass are the sustainability and eco-friendly characteristics of microalgae production and the feasibility to control the amount and type of favorable fatty acids in the oil. The main weakness so far relies on production scalability issues.

Considering microalgae biomass and lipids accumulation, the relationship between lubricant properties, chemical structure (FAs type) and application, a general guide for determining prospective microalgae oils for lubricants is suggested. The guide is based on the following steps: microalgae oil selection, qualitative prediction of main lubricant properties, precise characterization of lubricant properties and determination of the biolubricant applicability, respectively. If the oil properties satisfy the requirements for the target application, this can be used as biolubricant as it is produced; if not, the oil must be improved by chemical conversion and/or aggregating additives.

Several microalgae strains and parameters to produce oils with appropriate FAs for biolubricants were successfully identified from literature, and through the guide proposed, different applications are suggested for each oil. Microalgae oils appear to be promising alternatives to replace both petroleum- and VOs-derived lubricants in the future. Nonetheless, some challenges for production and use must be faced to guaranty a reliable supply chain. Among the most important challenges are the scalability of microalgae biomass production and oil extraction processes, continuous availability of the product with the required lubricant properties for the specific application without loss of the biodegradability rates.

## Authors contributions

L.I.F.-C.: Conceptualization; investigation; writing—original draft preparation; writing—review and editing; formal analysis; data curation; project administration. M.F.-M.: conceptualization; investigation; writing—original draft preparation; writing—review and editing; formal analysis; data curation. A.G.-S.: investigation; writing—review and editing. J.P.-G.: investigation; writing—review and editing; formal analysis; data curation. B.M.M.-S.: investigation; writing—review and editing; data curation. All authors have read and agreed to the published version of the manuscript.

## Figures and Tables

**Table 1:** Stress conditions to promote TAG microalgae cell accumulation.

Stress Condition	Effect of the Stress Condition on Growth and Lipids Accumulation	References
Deprivation of nutrients	Deprivation of nitrogen and phosphorus promote larger lipid accumulation in microalgae cells.	[107,108,109,110]
Oxygen saturation	Higher accumulation of dissolved oxygen in the culture broth promotes lipid degradation and decreases biomass productivity. So, it is important to regulate oxygen accumulation to enhance lipids increase.	[87,111]
Light intensity, illuminated/dark cycles and CO_2_ supply	Supply of appropriate light intensity and illuminated/dark cycles improves growth of different species of microalgae and lipid accumulation concomitant with CO_2_ supply at different concentrations.	[110,112,113,114]
Salinity	Salinity stimulates the accumulation of lipids in microalgae and avoids cell damage.	[115,116,117,118]
Temperature	Temperature changes promote enhancement in biomass productivity and carbon precursors for lipid hyper accumulation.	[119]
Mutant genes	Application of mutant genes increases lipid accumulation in microalgae cells due to changes in its metabolism.	[120]

**Table 2:** Description, advantages and disadvantages of methods used for harvesting microalgae biomass.

Type	Method	Description	Examples	Advantage	Disadvantage	References
Chemical	Flocculation/ Coagulation	Separation by interaction between negative charged cells and flocculant ions producing scattered units that settling down.	- Inorganic flocculants: metallic salts (Fe_2_(SO_4_)_3_, FeCl_3_, Al_2_(SO_4_)_3_, AlCl_3._ - Inorganic polymers: polyelectrolyte, polyaluminum.	- Fast and highly efficient separation.	- No green. - Contamination of downstream process.	[123,124,125]
Physical	Centrifugation	Separation by centrifugal force.	- Solid bowl decanter. - Solid ejecting disc. - Hydro-cyclone. - Solid bowl decanter. - Nozzle type.	- Efficient for microalgal size cells around - 3–30 µm. - Green	- Significant energy consumption. - Possible cell disruption by shear stresses.	[123,126,127]
	Filtration	Separation by using a filter medium through which only the fluid can pass.	- Microfiltration. - Macrofiltration. - Ultrafiltration. - Vacuum filtration. - Pressure filtration. - Tangential flow filtration.	- Green. - Efficient for large microalgae sizes.	- Not suitable for microalgae with size as *Chlorella* - *Dunaliella* - *Sncenedesmus.* - *Fouling/clogging phenomenon.* - *High operational cost.*	[124,127,128]
	Sedimentation	Separation by the action of gravity leaving a supernatant.	- The sedimentation rate depends on the algae genera. For example: - 0.2 m d^−1^ for diatoms. - 0.0–0.5 m d^−1^ for Cyanobacteria. - 0.1 m d^−1^ for green algae.	- Green. - Cost-effective due to low energy consumption.	- Depends on concentration of microalgae. - Low microalgae mass cannot not be effectively harvested.	[123,129]
	Flotation	Separation by using a thick foamed bed from which the solids are recovered by skimming.	- Dissolved air flotation. - Dispersed air floatation. - Electric flotation. - Ozonation dispersed flotation.	- Green. - Efficient for small size microalgae. - Small space required.	- Not appropriate for full-scale application.	[123,128]
	Electrical methods	Separation by application of an electric field to the microalgae cells with metallic electrodes.	- Electrophoresis. - Electroflotation. - Electroflocculation.	- Green. - Low toxicity compared to flocculation. - Low energy consumption compared to centrifugation.	- Low current density decreases flocculation.	[123,128]
Biological	Bioflocculation Autobioflocculation	Separation by the addition of micro- or macro-organisms to induce extra cellular polymer substances that promote flocculation through changes in pH, carbon sources supply, etc.	- Bacterial flocculation. - Fungal flocculation. - Actinomycetes flocculation. - Plant-based flocculation. - Algal-bacterial.	- Green. - Low-cost.	- Complex control of the procedure.	[123,128,130,131]

**Table 3:** Oil extraction methods from microalgae biomass.

Oil Extraction Technique	Description	Advantages	Disadvantage	References
Organic Solvents	Use of non-polar solvents to disrupt interactions between non-polar/neutral lipids.	- Inexpensive. - Solvent choice depends on the microalgae strain. - Specific for neutral lipids.	- No green. - Health and environmental risks. - Incompatibility of some solvents are with wet biomass.	[121,132]
Soxhlet extraction/Bligh and Dyer’s method	Use of hexane/mixture of chloroform and methanol as solvent to disrupt interactions between non-polar/neutral lipids.	- No solvent wastes. - Bligh and Dyer method yield extraction ≥95%.	- No green. - Health and environmental risks. - Incompatible with wet biomass.	[121]
Ionic liquids	Use of ionic solvents to disrupt microalgae cells and extract lipids.	- Low volatility. - Disrupt wet biomass under mild conditions.	- No green. - Changes in viscosity at low temperatures could affect lipid yield.	[134]
Supercritical fluids	Use of supercritical fluids as solvents to extract lipids.	- Green. - Safe for the environment. - Avoid flammable organic solvents.	- High operational cost. - Excess of water in biomass avoid diffusion transfer of the fluid.	[135]
Mechanical process	Use of mechanical methods as bead beating, pressing, ultrasonic-assisted extraction, electroporation, etc. for cell disruption and simultaneous oil extraction	- Green. - Applicable for wet biomass avoiding draying techniques. - Efficient lipid extraction. - Effective for pilot-scale and commercial levels. - Less dependence on the type of microalgae species to be processed.	- Possible contamination of lipids with cell debris. - Usually require higher energy inputs than the chemical or enzymatic methods. - Heat generation during mechanical disruptions may damage the final products.	[136]

**Table 4:** Chemical characteristics of bio-oil factors affecting main lubrication properties.

Property	Requirement	Chemical Factor	Characteristics	References
Lubricity	Low friction and wear under boundary lubrication.	Carbon chain length	Long (*n* ≥ 9) and linear (SFA, MUFA)	[138,139]
		FAs	Low unsaturation degree (SFA, MUFA)	[140,141]
		Polarity	High	[139,142,143]
Viscosity	Low viscosity	Carbon chain length	Short (viscosity increases with chain length)	[144,145]
		FAs	High unsaturation degree (PUFA)	[146]
	High viscosity	Carbon chain length	Long (viscosity increases with chain length)	[144,145]
		FAs	Low unsaturation degree (SFA, MUFA)	[146]
Pour point	Low pour point	FAs	High unsaturation degree (PUFA)	[144,147]
Oxidation stability	High oxidation stability	FAs	Low unsaturation degree (SFA, MUFA)	[148]

**Table 5:** Chemical additives for biolubricants formulation.

Additive	Function	Reported Effective Chemical Additives for Bio-Lubricants	References
Antioxidants	Interrupt or prevent the oxidation process without modifying other lubricant properties required. The process occurs in different ways depending on the structure and antioxidant mechanism.	Tocopherol, propyl gallate, l-ascorbic acid 6-palmitate, synthetic antioxidants (4,4′-methylenebis (2,6-di-tert-butylphenol), zinc diamyl dithiocarbamate, butylated hydroxy toluene, alkylated phenol/dithiophosphoric acid ester/diphenylamine and acylated chitosan schiff	[173,174,175]
Detergents and dispersants	Prevent the accumulation of sludge particles or other oil-insoluble substances. by dispersing and keeping them suspended in the oil.	Metal sulfonate, ash-less sulfonate, over based sulfonate, salicylates, alkyl phenolates, overbased carboxylate, polyisobu-tylene succinimides, glycidol modified succinimides, Mannich adducts, polyethylene glycol esters, polyol poly- (12-hydroxy stearic acid), piperazine derivatives, butyl citrate and ethyl oleate.	[176,177]
Viscosity modifiers	Provide the bio-lubricant with the viscosity magnitude required for both low and high temperatures keeping appropriate lubricity.	Olefin copolymer, ethylene-vinyl acetate (EVA) copolymer, olymethacrylates, styrene-diene copolymers and styrene-ester copolymers.	[178,179,180,181]
Pour point depressants	Limit the formation of large crystals during solidification process to provide oil flow at low temperatures.	Polymethacrylate backbone with a certain type of branching, 2-ethylhexyl oleate, isobutyl oleate, trimethylolpropane trioleate, pentaerythritol tetraoleate, diisodecyl adipate and Mannosylerythritol lipid.	[182,183,184]

**Table 6:** Compilation of data from prospect microalgae strains producing oils for lubricants.

Strain	Biomass Productivity (g L^−1^ h^−1^)	g Lipids/100 g DW	g Fatty Acid/100 g Lipids	Fatty Acids Profileg x-Fatty Acid/100 g Lipids	Photobioreactor/Illumination	Operational Conditions	Medium	Oil Extraction Method	References
*B. braunii* (UTEX LB 572)	0.18–0.22	14.72–15.64	7.26–11.69 SFA 63.10–82.9 MUFA 9.45–25.21 PUFA	(C4:0) 0.37–0.46 (C12:0) 0.20 (C14:0) 0.20–0.68 (C16:0) 6.40–9.36 (C16:1w7) 0.17–0.32 (C17:1w5) 1.22–2.68 (C18:0) 0.56–1.27 (C18:1w9c) 59.55–81.79 (C18:2w6c) 2.26–7.00 (C18:3w3) 5.27–11.20 (C20:1w9) 0.45–0.55 (C20:3w3) 1.21 (C20:4w6) 0.71–0.92 (C20:5w3) 1.56–2.23 (C22:1w9) 0.28 (C22:6w3) 3.87–3.88	Erlenmeyer flasks 170 μEm^−2^ s^−1^.	12 h light:12 h dark. 25 °C	CHU-medium, (g L^−1^) (0.2 KNO_3_; 0.04 K_2_HPO_4_; 0.08 CaCl_2_·2H_2_O; 0.1 MgSO_4_·7H_2_O; 0.1 C_6_H8O_7_·H_2_O; 0.1 C_6_H_5_FeO_7_·xH_2_O)	Chloroform-methanol (1:2)	[191]
*B. braunii* (IBL-C117)	0.10–0.15	7.037–7.822	17.30–20.46 SFA 21.95–65.60 MUFA 15.01–60.75 PUFA	(C4:0) 1.28–4.01 (C8:0) 0.47 (C12:0) 0.66 (C14:0) 0.61–1.26 (C16:0) 12.02–17.15 (C16:1w7) 0.52–1.13 (C17:0) 0.32 (C18:0) 0.74–1.26 (C18:1w9c) 14.78–24.60 (C18:2w6c) 4.63–9.36 (C18:3w3) 10.38–19.54 (C20:1w9) 0.90–1.30 (C20:5w3) 2.46 (C22:1w9) 23.34–49.35 (C22:6w3) 34.78	Erlenmeyer flasks 170 μEm^−2^ s^−1^.	12 h light:12 h dark. 25 °C			[191]
*B. terribilis* (IBL-C115)	0.10–0.18	15.33–16.79	9.91–10.60 SFA 71.66–83.56 MUFA 6.53–17.85 PUFA	(C4:0) 0.49–2.98 (C14:0) 0.34–0.64 (C16:0) 5.71–8.25 (C16:1w7) 0.23 (C17:0) 0.27 (C17:1w5) 0.70–2.35 (C18:0) 0.87–1.28 (C18:1w9c) 69.31–81.77 (C18:2w6c)1.92–5.84 (C18:3w3) 3.49–9.66 (C20:1w9) 0.66–0.69 (C20:4w6) 0.28–0.59 (C20:5w3) 0.84–1.77 (C22:1w9) 0.41 (C22:6w3) 2.26	Erlenmeyer flasks 170 μEm^−2^ s^−1^.	12 h light:12 h dark. 25 °C			[191]
*Chlorella* sp. 800	0.020	25.20	2.46 SFA 2.64 MUFA 2.64 PUFA	(C16:0) 1.86 (C16:1) 0.055 (C18:0) 0.60 (C18:1ω9) 2.59 (C18:2ω6) 1.97 (C18:3ω3) 0.67	2 L glass cylinder photobioreactors 200 μmol E m^−2^ s^−1^.	25 °C air enriched with 2% (*v*/*v*) CO_2_	Modified Tamiya medium (g L^−1^) (KNO_3_, 2.5; KH_2_PO_4_, 0.625; MgSO_4_·7H_2_O, 1.25; FeSO_4_·7H_2_O, 0.0045; Na_2_EDTA, 0.0186; and trace elements solution, 0.5 mL L^−1^). The trace elements solution (g L^−1^): H_3_BO_3_, 2.86; MnCl_2_·4H_2_O, 1.81; ZnSO_4_·7H_2_O, 0.222; NH_4_VO_3_, 0.023; and MoO_3_, 0.018.	Chloroform/methanol (2:1) and ball mill	[192]
*Chlorella saccharophila* 477	0.008	27.6	1.91 SFA 1.905 MUFA 4.23 PUFA	(C16:0) 1.56 (C16:1) 0.045 (C18:0) 0.35 (C18:1ω9) 1.86 (C18:2ω6) 4.04 (C18:3ω3) 0.19	2 L glass cylinder photobioreactors containing 200 μmol E m^−2^ s^−1^.	25 °C air enriched with 2% (*v*/*v*) CO_2_			[192]
*Chlorella minutissima* 494	0.016	NR	1.216 SFA 0.217 MUFA 2.3 PUFA	(C16:0) 1.18 (C16:1) 0.037 (C18:0) 0.036 (C18:1ω9) 0.18 (C18:2ω6) 2.05 (C18:3ω3) 0.25	2 L glass cylinder photobioreactors containing 200 μmol photons m^−2^ s^−1^.	25 °C air enriched with 2% (*v*/*v*) CO_2_			[192]
*Chlorella* sp. 313	0.018	NR	1.12 SFA 0.192 MUFA 1.6 PUFA	(C16:0) 1.08 (C16:1) 0.032 (C18:0) 0.04 (C18:1ω9) 0.16 (C18:2ω6) 1.41 (C18:3ω3) 0.19	2 L glass cylinder photobioreactors containing 200 μmol photons m^−2^ s^−1^.	25 °C air enriched with 2% (*v*/*v*) CO_2_			[192]
*Chlorella minutissima* 444	0.016	NR	1.04 SFA 0.18 MUFA 2.18 PUFA	(C16:0) 1.00 (C16:1) 0.04 (C18:0) 0.04 (C18:1ω9) 0.14 (C18:2ω6)1.95 (C18:3ω3) 0.23	2 L glass cylinder photobioreactors containing 200 μmol E m^−2^s^−1^.	25 °C air enriched with 2% (*v*/*v*) CO_2_			[192]
*Schizochytrium* sp.	0.537	49.53	44.81 PUFA	(C22:6) 100	1.500-L fermenter.	Control aeration 24 and 36 m^3^ h^−1^	40 g L^−1^ glucose and 0.4 g L-1 yeast extract dissolved in artificial sea water.	High-pressure homogenizer and n-hexane/ethanol (2:1).	[193]
*Schizochytrium* sp. HX-308	0.479	69.98	32.5 SFA 59.58 PUFA	(C14:0) 9.35 (C16:0) 23.15 (C22:5) 17.94 (C22:6) 41.64	10 L fermenter.	25 °C fed-batch cultivation.	Artificial sea water. (g/L): Na_2_SO_4_ 10; (NH_4_)_2_SO_4_ 0.8; KH_2_PO_4_ 4; KCl 0.2; MgSO_4_ 2; Monosodium glutamate 20 (g L^−1^); CaCl_2_ 0.1 (g L^−1^) and the trance elements (g/L): Na_2_EDTA 6, FeSO_4_ 0.29, MnCl_2_. 4H_2_O 0.86, ZnSO_4_ 0.8, CoCl_2_.6H2O 0.01, Na_2_MoO_4._2H_2_O 0.01, NiSO_4_.6H_2_O 0.06 and CuSO_4_ 5H_2_O 0.6.		[193,194]
*Phaeodactylum tricornutum*	0.043	NR	4.9 SFA 2 MUFA 5.6 PUFA	(C14:0) 0.7 (C16:0) 2.7 (C16:1) 2 (C16:3) 0.6 (C18:0) 1.5 (C20:5 n-3) 5	Flat panel airlift 200–1000 µmol E m^−2^ s^−1^).	1.25% (*v*/*v*) CO_2_ 16 h light per day 20 °C during the light period and 12–14 °C during the dark period.	Modified Mann and Myers medium (g L^−1^) 10 NaCl, 1.8 KCl, 2.4 MgSO_4_·7 H_2_O, and different N source urea, KNO_3_, NH_4_Cl,	NR	[195]
*Schizochytrium* sp.	1.25	68.6	20.69-34.32 SFA 0.43-1.88 MUFA 53.23-64.54 PUFA	(C14:0) 5.24–9.96 (C14:1) 0.43–1.88 (C16:0) 14.94–22.85 (C18:0) 0.51–1.51 (C22:5, n-6) 15.2–20.96 (C22:6, n-3) 38.03–43.58	50 L porous membrane-impeller bioreactor.	30 °C	Glucose (70 g L^−1^) and yeast extract. (8 g L^−1^) in artificial seawater.	Petroleum ether/diethyl ether (9:1) and methanol.	[196]
*Chlorella vulgaris*	0.005	30	38.8 SFA 45.4 MUFA 13.3 PUFA	(C16:0) 32.3 (C16:1) 17.7 (C18:0) 6.5 (C18:1) 27.7 (C18:2) 10.2 (C18:3) 3.1	5 L cylindrical algal photobioreactor 8-28 μmol photons m^−2^ s^−1^.	air sparger 30 °C light/dark period was 16/8 h	Waste industrial cane molasses. Gloucosan corn industry.	Bligh and Dyer method	[197]
*Dunaliella salina*	0.068	19.04	37.8 SFA 41.2 MUFA 20.9 PUFA	(C16:0) 15.37 (C16:1) 3.80 (C18:0) 7.15 (C18:1) 37.48 (C18:2)15.40 (C18:3) 2.31	10 L working bubble column photobioreactors 150 klux light intensity.	25 °C	Modified Guillard f/2 medium (without silica). Supplemented with NaNO_3_ (225 mg L^−1^) and sodium acetate (4 g L^−1^).	Ultrasound bath	[198]
*Nannochloropsis gaditana*	0.0036–0.0078	14.72–11.66	31.17 SFA 30.27 MUFA 20.79 PUFA	(C18:3 ω) 3.72 (C18:2) 17.07 (C18:1) 23.24 (C18:0) 8.99 (C16:1) 7.03 (C16:0) 22.18	4 L tank photo bioreactors 150 klux light intensity for 7 days.	24 °C sterile air at 1.4 L.min^−1^	Modified Guillard f/2 medium (without silica). 6 g L^−1^ of acetate and 225 mg L^−1^ of nitrate.	Modified Folch method and ultrasound, with a mixture of chloroform: methanol (3:1).	[199]

**Table 7:** Possible biolubricant applications of microalgae oils.

Microalgae Strain	Main Fatty Acids	Main Lubricant Properties *				Possible Applications
		Lubricity	Viscosity	Pour Point	Oxidation Stability	
*B. braunii* (UTEX LB 572)	MUFA (C:18)	✓✓✓	✓✓	✓	✓✓✓	Engine oil, gear oil, grease, metalworking fluid, insulating oil, refrigeration compressor oil, air mist lubricant, rock drill oil, vacuum pump oil, etc.
*B. braunii* (IBL-C117)	MUFA (C:22)	✓✓✓	✓✓✓	✓	✓✓✓	Engine oil, gear oil, grease, metalworking fluid, insulating oil, refrigeration compressor oil, air mist lubricant, rock drill oil, vacuum pump oil, etc.
*B. terribilis* (IBL-C115)	MUFA (C:18)	✓✓✓	✓✓	✓	✓✓✓	Engine oil, gear oil, grease, metalworking fluid, insulating oil, refrigeration compressor oil, air mist lubricant, rock drill oil, vacuum pump oil, etc.
*Chlorella* sp. 800	MUFA PUFA (C:18)	✓✓	✓✓	✓✓	✓✓	Concrete demolding oil, chainsaw oil, gear oil, grease, metalworking fluid, air mist lubricant, rock drill oil, etc.
*Chlorella saccharophila* 477	PUFA (C:18)	✓	✓	✓✓✓	✓	Concrete demolding oil, hydraulic fluid, chainsaw oil, air mist lubricant, etc.
*Chlorella minutissima* 494	PUFA (C:18)	✓	✓	✓✓✓	✓	Concrete demolding oil, hydraulic fluid, chainsaw oil, air mist lubricant, etc.
*Chlorella* sp. 313	PUFA (C:18)	✓	✓	✓✓✓	✓	Concrete demolding oil, hydraulic fluid, chainsaw oil, air mist lubricant, etc.
*Chlorella minutissima* 444	PUFA (C:18)	✓	✓	✓✓✓	✓	Concrete demolding oil, hydraulic fluid, chainsaw oil, air mist lubricant, etc.
*Schizochytrium* sp.	PUFA (C:22)	✓	✓	✓✓✓	✓	Concrete demolding oil, hydraulic fluid, chainsaw oil, air mist lubricant, etc.
*Schizochytrium* sp. HX-308	PUFA (C:22)	✓	✓	✓✓✓	✓	Concrete demolding oil, hydraulic fluid, chainsaw oil, air mist lubricant, etc.
*Phaeodactylum tricornutum*	PUFA (C:20)	✓	✓	✓✓✓	✓	Concrete demolding oil, hydraulic fluid, chainsaw oil, air mist lubricant, etc.
*Chlorella vulgaris*	MUFA (C:16)	✓✓✓	✓✓	✓	✓✓✓	Engine oil, gear oil, grease, metalworking fluid, insulating oil, refrigeration compressor oil, air mist lubricant, rock drill oil, vacuum pump oil, etc.
*Dunaliella salina*	MUFA (C:18)	✓✓✓	✓✓	✓	✓✓✓	Engine oil, gear oil, grease, metalworking fluid, insulating oil, refrigeration compressor oil, air mist lubricant, rock drill oil, vacuum pump oil, etc.
*Nannochloropsis gaditana*	SFA (C:18)	✓✓✓	✓✓	✓	✓✓✓	Engine oil, gear oil, grease, metalworking fluid, insulating oil, refrigeration compressor oil, air mist lubricant, rock drill oil, vacuum pump oil, etc.

* ✓ Low; ✓✓ Regular; ✓✓✓ High.

## Data Availability

Not applicable.

## Abbreviations

VO	Vegetable oil
MO	Mineral oil
SO	Synthetic oil
CoF	Coefficient of friction
PAO	Poly-alpha-olefin
HRAP	High-rate algal pond
ATP	Adenosine triphosphate
TAG	Triacyclglycerol
FA	Fatty acid
PUFA	Polyunsaturated fatty acid
MUFA	Monounsaturated fatty acid
SFA	Saturated fatty acid
BL	Boundary lubrication
